# The inland water macro-invertebrate occurrences in Flanders, Belgium

**DOI:** 10.3897/zookeys.759.24810

**Published:** 2018-05-22

**Authors:** Rudy Vannevel, Dimitri Brosens, Ward De Cooman, Wim Gabriels, Frank Lavens, Joost Mertens, Bart Vervaeke

**Affiliations:** 1 Flanders Environment Agency (VMM), Dr. De Moorstraat 24-26, B-9300 Aalst, Belgium; 2 Research Institute for Nature and Forest (INBO), Havenlaan 88 bus 73, B-1000 Brussels, Belgium

**Keywords:** Biotic indices, Flanders Environment Agency (VMM), macro-invertebrates, sediments, water quality

## Abstract

The Flanders Environment Agency (VMM) has been performing biological water quality assessments on inland waters in Flanders (Belgium) since 1989 and sediment quality assessments since 2000. The water quality monitoring network is a combined physico-chemical and biological network, the biological component focusing on macro-invertebrates. The sediment monitoring programme produces biological data to assess the sediment quality. Both monitoring programmes aim to provide index values, applying a similar conceptual methodology based on the presence of macro-invertebrates. The biological data obtained from both monitoring networks are consolidated in the VMM macro-invertebrates database and include identifications at family and genus level of the freshwater phyla Coelenterata, Platyhelminthes, Annelida, Mollusca, and Arthropoda. This paper discusses the content of this database, and the dataset published thereof: 282,309 records of 210 observed taxa from 4,140 monitoring sites located on 657 different water bodies, collected during 22,663 events. This paper provides some background information on the methodology, temporal and spatial coverage, and taxonomy, and describes the content of the dataset. The data are distributed as open data under the Creative Commons CC-BY license.

## Origin and context of the observations

### Biotic indices and monitoring networks

The macro-invertebrates dataset contains data on the occurrence of macro-invertebrates in inland water bodies, obtained from water and sediment quality assessments. The objective of the assessments is to provide biotic index values. Biotic indices are based on two properties: the number of taxa indicating the biodiversity, and the sensitivity of organisms (of a selected number of taxa) to (mainly organic) pollution. Biotic indices based on macro-invertebrates complement the physico-chemical water quality and are commonly considered as pollution indicators (hence they are called water quality indices), although they also partially reflect overall habitat conditions. Higher biotic index values tend to reflect the general water body or habitat status; lower values indicate the water quality conditions, of which the oxygen level prevails. Since biotic indices are intended for quick and practical use, numbers of organisms are estimated and the identification level is restricted.

The biological water-quality monitoring programme based on macro-invertebrates was initiated by Belgian national legislation in 1987. It was further developed in the 1990s by the Flemish authorities responsible for water quality control of surface waters, to meet regional environmental legislation. After 2000, it was redirected in support of the Water Framework Directive (WFD, 2000/60 EC). The water quality monitoring network is developed by the Flanders Environment Agency (VMM, https://www.vmm.be/) and its preceding Flemish water authorities involved in wastewater and sanitation to assess the quality status of water bodies in Flanders. The quality is assessed by means of biotic indices: the Belgian Biotic Index (BBI) (De Pauw and Vanhooren 1983; De Pauw and Vannevel 1991; NBN 1984) and the Multimetric Macroinvertebrate Index Flanders (MMIF) (Gabriels et al. 2010). These indices aim to assess water quality of (mainly impacted) public waters on an annual or multi-annual basis in different water types of both freshwater and brackish aquatic systems. Related biological quality elements include diatoms, macrophytes, and fish.

The sediments quality-monitoring network was developed in 2000 to support decision-making on the dredging of waterways. Between 1992 and 2000, sediment monitoring was part of research programmes of which the results are also included in the dataset. The macro-invertebrates are one element of the Sediment Quality index, completing information on sediments quantity and substrate composition. The methodology is described by De Deckere et al. (2000), De Pauw et al. (2000), and De Pauw and Heylen (2001), and deals with sediment characterization by means of the TRIAD approach (AMINAL 1998). This threefold approach combines a biological, ecotoxicological and physical-chemical component.

The VMM water quality database contains data on occurrence, abundance, habitat conditions, pollutants, quality indices, and sampling conditions. Iconographic material is available, but not included in the published dataset. Regular sampling at a regional scale is ongoing since 1989. The database is modelled according to the field protocol and identification list published by De Pauw and Vannevel (1991), the forms being regularly updated and adapted by VMM’s monitoring staff. The database was designed and developed in 2000 to allow automated calculation of biotic index values. Biotic indices are used to produce statistics and maps on ecological water quality: the BBI (range: 0–10) was used for VMM’s annual reports from 1990 onwards and replaced by the MMIF (range: 0–1) in 2011. The MMIF allows the calculation of Ecological Quality Ratios (EQRs) as required by the EU Water Framework Directive (2000/60/EC), along with other biological and physico-chemical quality elements. Annual VMM reports are available online (http://www.vmm.be/publicaties).

The data elements of the VMM sediment quality database are similar to those of the water quality database. Iconographic material is also available, although not publicly accessible. Regular sampling at a regional scale started in 2000, with the database being modelled according to the field protocol and identification list published by De Deckere et al. (2000). The database was designed and developed in 2000 to allow automated calculation of the Biotic Sediment Index (BSI; range: 0–10) values, in order to produce statistics and maps on sediments water quality. Annual sediment reports are published from 2000 onwards (in Dutch) and are available online (http://www.vmm.be/publicaties).

In total, the dataset covers the period between 1989 and 2016, and contains 282,309 records of 210 observed taxa from 22,663 samples of 4,140 monitoring sites located on 657 different water bodies.

### Spatial and temporal coverage

Belgium is located in the centre of Western Europe and its surface area reaches 30,528 km². Of the three administrative regions, Flanders is situated in the northern part and covers an area of 13,522 km² (which is 44.29 % of the Belgian territory), roughly situated at 51° latitude and 4° longitude. Flanders has a temperate maritime climate that is strongly influenced by the North Sea and the Atlantic Ocean. Average precipitation in Belgium is 852 mm during the period 1981–2010, with the highest value (1089 mm) in 2001 and the lowest value (640 mm) in 1989. Mean temperature between 1981 and 2010 is 10.5 °C. Population density in Flanders is 462 inhabitants/km² in 2010, which makes it one of the most densely populated areas in Europe.

**Figure 1.**
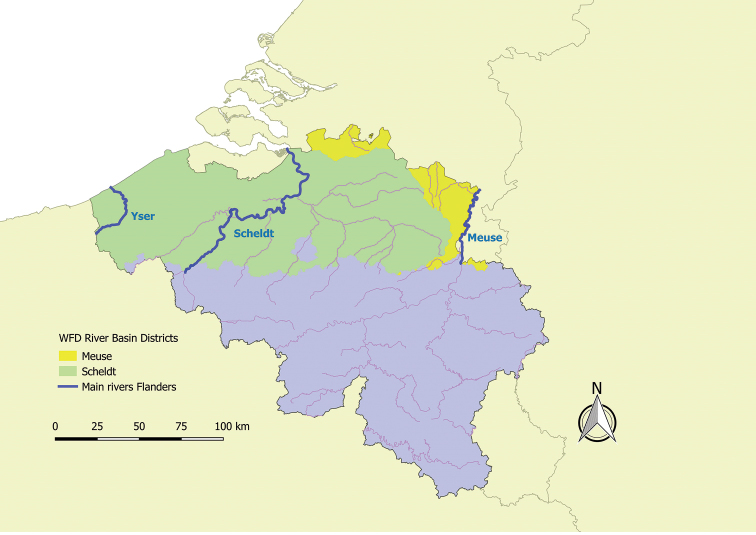
Location of the region of Flanders in Belgium, its main rivers (Yser, Scheldt, Meuse) and the subunits of the WFD river basin districts.

The three major rivers in Flanders are the Yser, Scheldt, and Meuse (Fig. 1). All discharge into the North Sea, but only the river Yser and a few canals drain directly into the sea within the Flemish territory. According to the WFD, the river Yser and Scheldt basins are part of the Scheldt River Basin District that also includes parts of France and the Netherlands. In a similar way, the Meuse River Basin District covers parts of Belgium, France, Luxemburg, Germany, and the Netherlands. Within the Belgian territory, both river basin districts are subdivided into so-called subunits according to the regions (Flanders, Wallonia, and the Brussels Capital Region). As such, the dataset covers the Scheldt-Flanders (BESCHELDE-VL) and the Meuse-Flanders (BEMAAS-VL) subunits. The WFD basin structure is based on clusters of 11 Flemish river basins delineated in the early 1990s.

Bounding box for covered area and period:

– Flanders:

• 50.68 to 51.51 N; 2.54 to 5.92 E (DD)

– Temporal coverage:

• Surface water quality: 1989-03-15 – 2016-11-30

• Sediment quality: 2000-07-12 – 2016-08-02

### Water quality monitoring network and methodology

**Figure 2.**
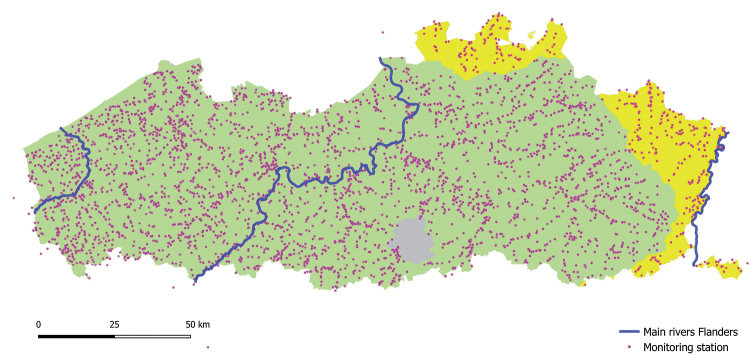
The VMM monitoring network for surface water quality assessments.

The water quality network (Fig. 2) comprises surface water bodies of all types: flowing and standing waters (including rivers, streams, canals, inner city waters); navigable and non-navigable waterways and watercourses; natural, heavily modified and artificial water courses and lakes; freshwater and transitional water bodies. See VMM (2009) for a description of the typological classification of the Flemish water bodies.

**Figure 3.**
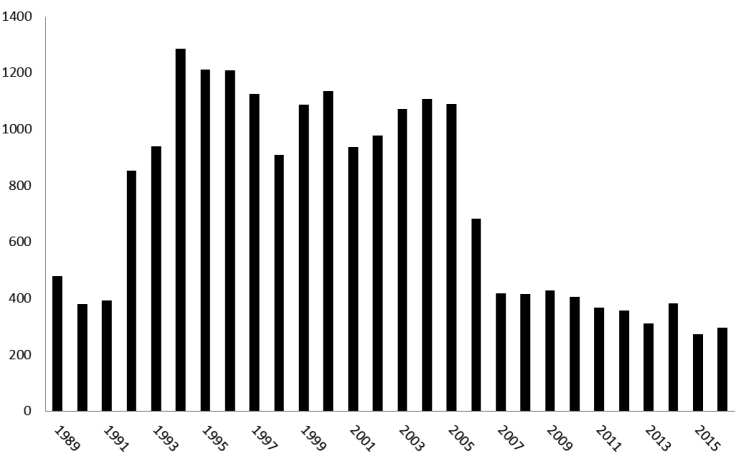
Sampling effort of the water quality network (number of samples per year).

Since 1989, over 4,106 locations in estuaries, rivers, streams, canals, and standing waters have been sampled by the VMM. Over the years, and in particular after the implementation of the Water Framework Directive in 2000, this network gradually evolved towards a set of about 500 potential monitoring stations, of which a core set of 39 are frequently monitored for macro-invertebrates. Monitoring efforts are highest between 1992 and 2005 (Fig. 3). Five monitoring stations are located in France and 17 in the Netherlands. Biological sampling is accompanied by general physico-chemical field measurements.

Since the monitoring strategy aims to assess water quality for decision-making, most sampling stations are located on pollution-impacted water bodies. Those water bodies include head streams, tributaries, and canals or waterways, as well as locations up- and downstream from significant pollution sources (e.g. industrial plants, wastewater treatment plants). Monitoring sites on smaller water bodies have been selected for specific impact assessments, e.g. eutrophication from farming. Occasionally, smaller investigations, often on request, resulted in the monitoring of protected areas, upper stream areas, oxbows, gravel pits, or ponds. The monitoring strategy has changed since the beginning of the 1990s, moving from a vast and dense network to a rather restricted core set of stations designed to meet European reporting obligations, in particular the WFD.

The monitoring process includes sampling and field observations (noted on a field protocol), treatment of the sample in the laboratory (sieving and sorting out organisms by handpicking), identification of the organisms, and derivation of the biotic index value from an index table. The procedure for sampling macro-invertebrates in surface waters is described by De Pauw and Vanhooren (1983) and NBN (1984) for handnet sampling (kicking method), and by De Pauw et al. (1986) for using artificial substrates. The sampling method was applied as follows: handnet (87%), artificial substrates (13%), unknown (less than 1%). Field observations, including in situ physico-chemical measurements, are noted on a field protocol. The methods for sampling and sample treatment are standardised and certified; additional information can be obtained by contacting the corresponding authors. Relevant field information is included in the reported database, partially aggregated in the Darwin Core term ‘dwc:dynamicProperties’. The geographic coordinates in the dataset indicate the precise location of the station on the river stretch sampled (‘dwc:locationID’). However, as the sampling method prescribes the sampling of different microhabitats at the location and over a length of several metres, the co-ordinates are indicative of a stretch up to about 10 metres.

Overall, the water quality database covers two river basin district subunits, 19 water body types, 656 water bodies located in Flanders, 4,106 monitoring stations (of which 22 are located outside the Belgian territory), 20,545 samples, 208 taxa, and 267,648 taxonomic identifications.

### Sediment quality monitoring network and methodology

**Figure 4.**
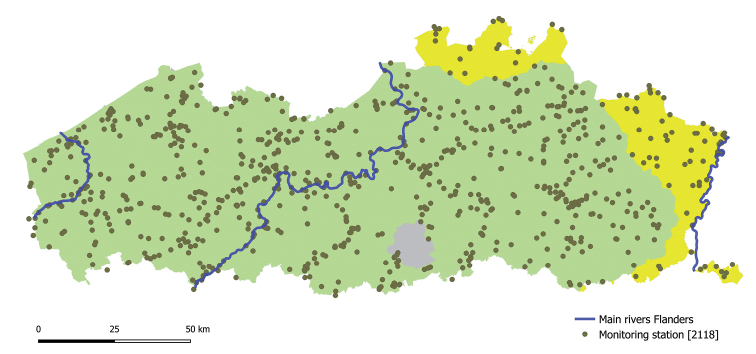
The VMM monitoring network for sediment quality assessments.

Sediment quality monitoring stations (Fig. 4) include mainly headwaters and navigable waterways for the purpose of dredging to secure shipment. Other sites are located on water bodies subject to sanitation. As such, sediment monitoring sites are located on rivers, canals and docks, including tidal rivers and locations in industrialised harbour areas.

**Figure 5.**
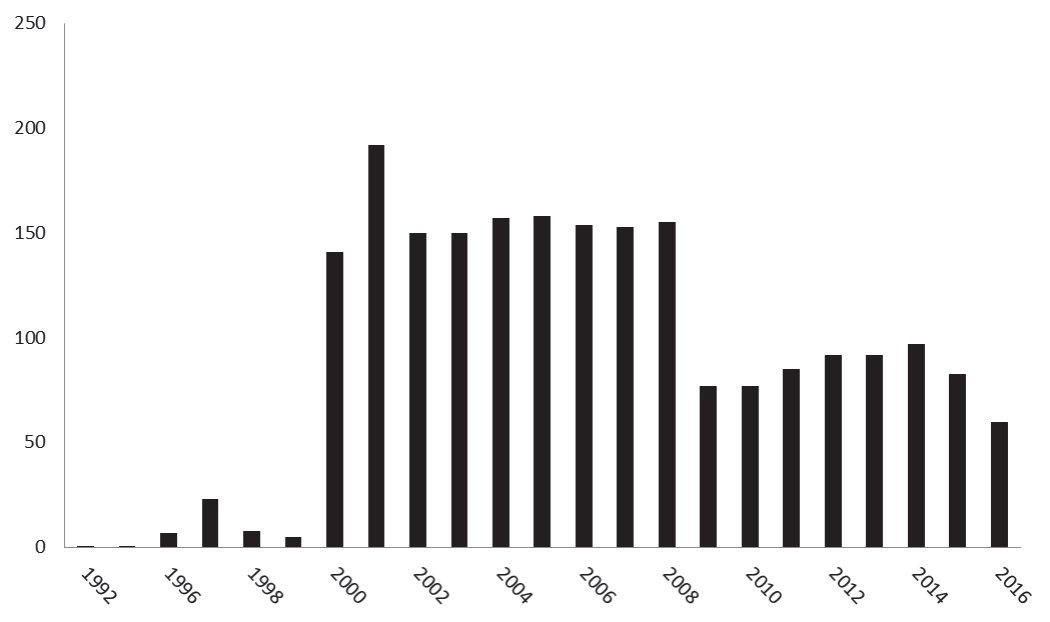
Sampling effort of the sediment quality network (number of samples per year).

Since 1992, over 805 locations in estuaries, rivers, and canals have been monitored by the VMM. According to the increase of knowledge and management requirements, this network gradually evolved towards a core set of about 300 monitoring stations, monitored with a sexennial frequency. Monitoring efforts are highest between 2000 and 2008 (Fig. 5). Two monitoring stations are located in France and nine in the Netherlands.

The process of sediment monitoring is similar to water quality monitoring, but some different practices are applied. The procedure for sampling macro-invertebrates in sediments is described in a compendium for sampling and analysis, produced by the Flemish ministry for nature and environment (AMINAL 1998). Prescribed sampling methods include core and grab samplers. The sampling methods were applied as follows: grab sampler (90%), core sampler (10%), or unknown (less than 1%). Field observations are noted on a field protocol. The methods for sampling and sample treatment are standardised and certified; additional information can be obtained by contacting the corresponding authors. Relevant field information is included in the reported database, partially aggregated in ‘dwc:dynamicProperties’. The geographic coordinates in the dataset indicate the precise location of the station on the river stretch investigated (‘dwc:locationID’). However, as the sampling method prescribes subsampling, the co-ordinates are indicative of a stretch up to about 50 metres.

Overall, the water sediment database covers 2 river basin district subunits, 19 water body types, 405 water bodies located in Flanders, 805 monitoring stations (of which 11 are located outside the Belgian territory), 2,118 samples, 150 taxa, and 14,661 taxonomic identifications.

### Quality Control

The following comments regarding quality control apply to both the water and the sediment quality network. Field protocols and identification lists were used to allow comparable observations between networks and samplers. Field observations are highly objective in this way, although differences resulting from personal interpretation may still occur. However, content slightly changed over the years since a number of protocol versions with new and adapted data elements have been produced. The same applies to taxonomic identification. In this respect, it is advised to consult also the expert publications based on the VMM dataset (see reference list: ‘Publications based on this dataset’). Differences with present taxonomic nomenclature can be experienced and, for this reason, distortion of biotic index values is avoided as much as possible by using a ‘fixed’ nomenclature. Be aware that a large number of people have contributed to the taxonomic identification over the years, which has unavoidably led to errors (misidentification, inclusion of terrestrial specimen, etc.). In this respect, the identifications of, for instance, Aeolosomatidae may require further examination. For taxonomic verification of sample material, experts should contact the Royal Belgian Institute of Natural Sciences (RBINS, Brussels) (http://collections.naturalsciences.be/) where the VMM macro-invertebrate samples are stored. RBINS identifiers (coded KBIN-IG: xxxxx) apply to all VMM samples of a single year.

### Additional information

The water quality and sediment networks are part of a broader monitoring programme, using the same monitoring locations. Additional information is available on other biological (including macrophytes and diatoms), habitat (hydromorphology, water level, and flow) and physico-chemical quality elements, according to the requirements of the European Commission (Water Framework Directive and other environmental legislation), European authorities (European Environment Agency), and other reporting obligations. Detailed information about these programmes can be obtained by contacting the authors. As far as the spatial data are aligned with the INSPIRE directive (2007/2/EC), the monitoring site identifier can be used to search related data. In the other cases, VMM (info@vmm.be) can be consulted. At the time of publication, no taxonomic data of this dataset have been reported to international authorities.

## Taxonomy

### Taxonomic coverage

Taxonomic information is restricted to a selected set of macro-invertebrate taxa, with identification limited to the taxonomic level required and needed to calculate biotic index values. For that purpose, taxa are listed in a protocol on which identifications are ticked off. The same identification lists apply to both the water and the sediment quality networks. It is mandatory to use the VMM identification lists to calculate biotic index values.

Taxonomic identification levels range from order to genus level and is the same for both the water quality and sediment methodology:

– For water quality monitoring, taxonomic groups and identification levels are defined by De Pauw and Vanhooren (1983) as follows: Platyhelminthes: genus; Oligochaeta: family; Hirudinea: genus; Mollusca: genus; Crustacea: family; Plecoptera: genus; Ephemeroptera: genus; Trichoptera: family; Odonata: genus; Megaloptera: genus; Hemiptera: genus; Coleoptera: family; Diptera: family, except Chironomidae (two groups: Chironomidae *forma* thummi-plumosus and Chironomidae *forma* non-thummi-plumosus); Hydracarina (presence).

– For sediment monitoring, taxonomic groups and identification levels are defined by De Pauw and Heylen (2001): Platyhelminthes: genus; Oligochaeta: (presence); Hirudinea: genus; Mollusca: genus; Crustacea: family; Plecoptera: genus; Ephemeroptera: genus; Trichoptera: family; Odonata: genus; Megaloptera: genus; Hemiptera: genus; Coleoptera: family; Diptera: family, except Chironomidae (two groups: Chironomidae *forma* thummi-plumosus, Chironomidae *forma* non-thummi-plumosus); Hydracarina (presence).

The identification list by De Pauw and Vannevel (1991) applied to the water quality network in the 1990s includes 220 taxa. At present, the VMM identification list contains 229 taxa reported from Flemish standing waters and watercourses. Differences in numbers of taxa are a result of:

– taxonomic classification, in particular the splitting of some genera of molluscs. As an example, the splitting of the genus *Physa* into the genera *Physa* and *Physella* is only clear from 2005 onwards;

– the inclusion of alien species (Gabriels et al. 2005), e.g. *Corbicula*, Ampharetidae (including *Hypania invalida*);

– the addition of freshwater-brackish species, mainly crustaceans, but also occasional records of Ampharetidae (including *Hypania invalida*).

In preparation of the dataset for inland water, a number of records have been included or excluded, which resulted in the publication of 226 taxa. These changes relate to:

– Included: brackish-marine taxa: Panopeidae, Varunidae (Crustacea), Sabellidae (Polychaeta), *Rangia* (Mollusca);

– Included, but not systematically recorded taxa: Nemathelminthes, Coelenterata (including *Hydra*), Hydracarina, Cladocera (including *Daphnia*), Ostracoda, Copepoda;

– Excluded: marine taxa: Veneroida (Mollusca);

– Excluded: non-macro-invertebrates (e.g. fish species);

– Excluded: occasionally recorded non-target taxa: Porifera, Bryozoa, Nemertea, Collembola.

A particular case concerns the chironomids, of which two ‘forms’ occur (thummi-plumosus and non-thummi-plumosus), referring to the presence of external respiratory tubes at low oxygen conditions. This distinction is of importance for calculating the biotic index. They are denoted in ‘dwc:taxonRemarks’ by Chironomidae thummi-plumosus and Chironomidae non-thummi-plumosus.

It is worth mentioning that a few scientists used the VMM samples for in-depth taxonomic research on species occurrence and distribution, with identification beyond the level indicated (see reference list: publications based on this dataset). Expert data are not included in the reported datasets. An example of such a continued study on VMM sample material is Boets et al. (2016) on alien macroinvertebrates in Flanders, with identification up to species level. These data are also available on GBIF: http://www.gbif.org/dataset/3c428404-893c-44da-bb4a-6c19d8fb676a. Unfortunately, due to the different design of the studies, the occurrence records of both datasets are not unambiguously connectable.

### Taxonomic ranks

The following list contains the taxonomic classification of macro-invertebrates that have been recorded, that are present, or that are expected to occur in Belgium. According to the VMM identification list, 245 taxa are selected. Out of this number, 210 taxa have been observed between 1989 and 2016. Some taxa (indicated with *) have been recorded in the past or are expected to occur in Belgium, but do not appear in the dataset. One reason is that, due to geographical differences, some taxa of Odonata, Ephemeroptera, and Plecoptera in particular are considered less common in Flanders. On the other hand, the dataset also includes some non-target taxa. Single-species taxa occurring within the investigated area are indicated by adding the species name, although the dataset only contains the genus name. The occurrence of single-species taxa was checked against the Dutch register of species (Nederlands Soortenregister, http://www.nederlandsesoorten.nl/).

**Kingdom**: Animalia

**Phylum**: Coelenterata, **Class**: Hydrozoa

**Species**: Hydra sp., Craspedacusta sowerbii*, Cordylophora caspia*

**Phylum**: Platyhelminthes, **Class**: Turbellaria, **Order**: Rhabdocoela

**Genus**: *Mesostoma*

**Phylum**: Platyhelminthes, **Class**: Turbellaria, **Order**: Seriata

**Family**: Planariidae, **Genera/species**: *Crenobia alpina*, Phagocata vitta*, *Planaria torva*, *Polycelis*

**Family**: Dugesiidae, **Genus**: *Dugesia*

**Family**: Dendrocoelidae, **Genera/species**: *Bdellocephala punctata*, *Dendrocoelum*

**Phylum**: Nematoda

**Phylum**: Nematomorpha

**Genus**: Gordius*

**Phylum**: Annelida, **Class**: Oligochaeta

**Families**: Aeolosomatidae, Branchiobdellidae, Enchytraeidae, Haplotaxidae, Lumbricidae, Lumbriculidae, Naididae, Tubificidae

**Phylum**: Annelida, **Class**: Polychaeta

**Families**: Ampharetidae, Sabellidae

**Phylum**: Annelida, **Class**: Hirudinea

**Family**: Piscicolidae, **Genera**: *Cystobranchus*, *Piscicola*

**Family**: Glossiphoniidae, **Genera/species**: *Glossiphonia*, *Helobdella*, *Hemiclepsis marginata*, *Placobdella* (syn. *Haementeria*) *costata*, *Theromyzon tessulatum*

**Family**: Hirudidae, **Genera/species**: *Haemopis*, Hirudo medicinalis*

**Family**: Erpobdellidae, **Genera**: *Dina*, *Erpobdella*, *Trocheta*

**Phylum**: Mollusca, **Class**: Gastropoda

**Family**: Neritidae, **Genus**: *Theodoxus*

**Family**: Viviparidae, **Genus**: *Viviparus*

**Family**: Valvatidae, **Genus**: *Valvata*

**Family**: Bithyniidae, **Genus**: *Bithynia*

**Family**: Hydrobiidae, **Genera/species**: Avenionia*, *Bythinella*, *Lithoglyphus naticoides*, *Marstoniopsis scholtzi*, *Potamopyrgus antipodarum*, *Pseudamnicola confusa*

**Family**: Physidae, **Genera/species** : *Aplexa hypnorum*, *Physa*, *Physella*

**Family**: Lymnaeidae, **Genera/species** : *Lymnaea*, *Myxas glutinosa*

**Family**: Planorbidae, **Genera/species**: *Anisus*, *Armiger crista*, *Bathyomphalus contortus*, *Gyraulus*, *Hippeutis complanatus*, *Menetus dilatatus*, *Planorbarius corneus*, *Planorbis*, *Segmentina nitida*

**Family**: Ancylidae, **Genera/species** : *Ancylus fluviatilis*, *Ferrissia*

**Family**: Acroloxidae, **Species**: *Acroloxus lacustris*

**Phylum**: Mollusca, **Class**: Bivalvia

**Family**: Margaritiferidae, **Species**: Margaritifera margaritifera*

**Family**: Unionidae, **Genera**: *Anodonta*, *Pseudanodonta*, *Unio*

**Family**: Dreissenidae, **Genera**: *Dreissena*, *Mytilopsis*

**Family**: Sphaeriidae, **Genera**: *Sphaerium*, *Pisidium*

**Family**: Cyrenidae, **Genus**: *Corbicula*

**Family**: Mactridae, **Genus**: *Rangia*

**Phylum**: Arthropoda, **Class**: Arachnida, **Order**: Aranea

**Species**: Argyroneta aquatica*

**Phylum**: Arthropoda, **Class**: Arachnida, **Order**: Actinedida (syn. Hydracarina)

**Phylum**: Arthropoda, **Subphylum**: Crustacea, **Class**: Branchiopoda, **Order**: Anostraca, Notostraca, Conchostraca

**Families/species**: Chirocephalidae*, Leptestheriidae: Leptestheria dahalacensis*, Limnadiidae: Limnadia lenticularis*, Triopsidae*

**Phylum**: Arthropoda, **Subphylum**: Crustacea, **Class**: Branchiopoda, **Order**: Cladocera

**Families**: Daphniidae a.o.

**Phylum**: Arthropoda, **Subphylum**: Crustacea, **Class**: Ostracoda

**Phylum**: Arthropoda, **Subphylum**: Crustacea, **Class**: Maxillopoda, **Subclass**: Copepoda

**Phylum**: Arthropoda, **Subphylum**: Crustacea, **Class**: Maxillopoda, **Subclass**: Branchiura

**Families**: Argulidae: *Argulus*

**Phylum**: Arthropoda, **Subphylum**: Crustacea, **Class**: Malacostraca, **Orders**: Mysidacea, Amphipoda, Isopoda, Decapoda

**Families**: Asellidae, Astacidae, Atyidae: *Atyaephyra desmaresti*, Cambaridae, Corophiidae: *Corophium curvispinum*, Crangonyctidae, Gammaridae, Janiridae, Mysidae, Palaemonidae, Panopeidae, Sphaeromatidae, Talitridae, Tanaidae, Varunidae: *Eriocheir sinensis*

**Phylum**: Arthropoda, **Class**: Insecta, **Order**: Ephemeroptera

**Family**: Siphlonuridae, **Genera**: Isonychia*, *Metreletus*, Siphlonurus*

**Family**: Baetidae, **Genera**: *Baetis*, *Centroptilum*, *Cloeon*, *Procloeon*

**Family**: Oligoneuriidae, **Genus**: Oligoneuriella*

**Family**: Heptageniidae, **Genera**: *Ecdyonurus*, Epeorus*, *Heptagenia*, Rhitrogena*

**Family**: Leptophlebiidae, **Genera**: Habroleptoides*, *Habrophlebia*, *Leptophlebia*, *Paraleptophlebia*

**Family**: Ephemerellidae, **Genus**: *Ephemerella*

**Family**: Potamanthidae, **Species**: Potamanthus luteus*

**Family**: Ephemeridae, **Genus**: *Ephemera*

**Family**: Polymitarcidae, **Species**: Ephoron virgo*

**Family**: Caenidae, **Genera/species**: *Brachycercus harisella*, *Caenis*

**Phylum**: Arthropoda, **Class**: Insecta, **Order**: Odonata

**Family**: Calopterygidae, **Genus**: *Calopteryx*

**Family**: Lestidae, **Genera**: *Lestes*, *Sympecma*

**Family**: Platycnemidae, **Species**: *Platycnemis pennipes*

**Family**: Coenagrionidae, **Genera/species**: *Cercion lindeni*, *Ceriagrion tenellum*, *Coenagrion*, *Enallagma cyathigerum*, *Erythromma*, *Ischnura*, *Nehalennia speciosa*, *Pyrrhosoma nymphula*

**Family**: Aeshnidae, **Genera/species**: *Aeshna*, *Anax*, *Brachytron pratense*

**Family**: Gomphidae, **Genera**: *Gomphus*, *Onychogomphus*, Ophiogomphus*

**Family**: Cordulegasteridae, **Species**: *Cordulegaster boltonii*

**Family**: Corduliidae, **Genera/species**: *Cordulia aenea*, Epitheca bimaculate*, Oxygastra curtisii*, *Somatochlora*

**Family**: Libellulidae, **Genera**: *Crocothemis erythrea*, *Leucorrhinia*, *Libellula*, *Orthetrum*, *Sympetrum*

**Phylum**: Arthropoda, **Class**: Insecta, **Order**: Plecoptera

**Family**: Taeniopterygidae, **Genera**: Brachyptera*, Rhabdiopteryx*, *Taeniopteryx*

**Family**: Nemouridae, **Genera**: Amphinemura*, *Nemura*, *Nemurella*, *Protonemura*

**Family**: Capniidae, **Genus**: Capnia*

**Family**: Leuctridae, **Genus**: *Leuctra*

**Family**: Perlidae, **Genera**: Dinocras*, Marthamea*, *Perla*

**Family**: Perlodidae, **Genera**: Isogenus*, *Isoperla*, Perlodes*

**Family**: Chloroperlidae, **Genus**: Chloroperla*

**Phylum**: Arthropoda, **Class**: Insecta, **Order**: Hemiptera

**Family**: Mesoveliidae, **Species**: *Mesovelia furcata*

**Family**: Hydrometridae, **Genera**: *Hydrometra*

**Family**: Hebridae, **Genus**: *Hebrus*,

**Family**: Veliidae, **Genera**: *Microvelia, Velia*

**Family**: Gerridae, **Genus**: *Gerris*

**Family**: Naucoridae, **Species**: *Ilyocoris cimicoides*, *Naucoris maculatus*

**Family**: Aphelocheiridae, **Species**: *Aphelocheirus aestivalis*

**Family**: Nepidae, **Species**: *Nepa cinerea*, *Ranatra linearis*

**Family**: Pleidae, **Species**: *Plea minutissima*

**Family**: Notonectidae, **Genus**: *Notonecta*

**Family**: Corixidae, **Genera**: *Arctocorisa*, *Callicorixa*, *Corixa*, *Cymatia*, *Glaenocorisa*, *Hesperocorixa*, *Micronecta*, *Paracorixa*, *Sigara*

**Phylum**: Arthropoda, **Class**: Insecta, **Order**: Neuroptera

**Families**: Sisyridae, Osmylidae

**Genera**: Sisyrus*, Osmylus*

**Phylum**: Arthropoda, **Class**: Insecta, **Order**: Megaloptera

**Families**: Sialidae

**Genus**: *Sialis*

**Phylum**: Arthropoda, **Class**: Insecta, **Order**: Coleoptera

**Families/species**: Dryopidae, Dytiscidae, Elminthidae, Georissidae: Georissus crenulatus*, Gyrinidae, Haliplidae, Hydraenidae, Hydrophilidae, Hygrobiidae: *Hygrobia hermanni*, Noteridae, Psephenidae: *Eubria palustris*, Scirtidae

**Phylum**: Arthropoda, **Class**: Insecta, **Order**: Trichoptera

**Families**: Beraeidae, Brachycentridae, Ecnomidae, Glossosomatidae, Goeridae, Hydropsychidae, Hydroptilidae, Lepidostomatidae, Leptoceridae, Limnephilidae, Molannidae, Odontoceridae, Philopotamidae, Phryganeidae, Polycentropodidae, Psychomyidae, Rhyacophilidae, Sericostomatidae

**Phylum**: Arthropoda, **Class**: Insecta, **Order**: Lepidoptera

**Genera/species**: Acentropus niveus*, Cataclysta*, Nymphula*, Parapoynx*

**Phylum**: Arthropoda, **Class**: Insecta, **Order**: Diptera

**Families**: Athericidae, Blephariceridae*, Ceratopogonidae, Chaoboridae, Chironomidae (formae *thummi-plumosus* and *non-thummi-plumosus*), Culicidae, Cylindrotomidae, Dixidae, Dolichopodidae, Empididae, Ephydridae, Limoniidae, Muscidae, Psychodidae, Ptychoderidae, Rhagionidae, Scatophagidae, Sciomyzidae, Simulidae, Stratiomyidae, Syrphidae-*Eristalinae*, Tabanidae, Thaumaleidae, Tipulidae.

## Dataset

### Dataset specifications

The occurrence dataset is available at:

IPT: https://data.inbo.be/ipt/resource?r=vmm-macroinvertebrates-events

GBIF: https://www.gbif.org/dataset/5ca32e22-1f1b-4478-ba7f-1916c4e88d67

Recommended citation to the dataset:

VMM, INBO (2018). Inland water macro-invertebrate occurrences in Flanders, Belgium. Flanders Environment Agency (VMM). Sampling_event Dataset https://doi.org/10.15468/4cvbka accessed via GBIF.org.

Specifications:

– Object name: *Inland water macro-invertebrate occurrences in Flanders, Belgium*

– Character encoding: UTF-8

– Format name: Darwin Core Archive format

– Format version: 1.8

– Distribution: https://ipt.inbo.be/archive.do?r=vmm-macroinvertebrates-events

– First Publication date of data: 2018-01-17

– Language: English

– Licenses of use: https://creativecommons.org/licenses/by/4.0/legalcode

– Metadata language: English

– Date of metadata creation: 2018-02-01

– Hierarchy level: Dataset

This is an open database distributed under the Creative Commons Attribution License (CC BY 4.0) which permits unrestricted use, distribution, and reproduction in any medium, provided the original work is properly cited. It is recommended, as far as possible, to notify the corresponding authors, especially when using data for scientific research.

### Dataset description

The occurrence data from the VMM macro-invertebrate database are extracted, standardised and published as a single sample-based dataset in Darwin Core Archives format. The dataset contains data on the surface water quality and the sediment quality networks of inland waters, since the data of both networks refer to the same locations, habitat specifications and taxonomic lists. Within the limits of the sampling procedure, sampling requires a maximum effort to obtain organisms, without guaranteeing taxonomic completeness of the whole biocoenosis. Verification of the origin of the data is possible on the basis of ‘dwc:habitat’, referring to ‘water body’ in the case of the water quality network and to ‘sediment’ in the case of the sediment quality network.

Sampling stations have a unique code referring to a single location, but represent one or several quality elements. The VMM station code links to other biological (diatoms, macrophytes) and physico-chemical monitoring programmes. Only physico-chemical field observations are included in the GBIF dataset in ‘dwc:dynamic properties’.

Concept and methodology of the Belgian Biotic Index (BBI) and the Biotic Sediment Index (BSI) are comparable. BBI, MMIF and BSI values are reported under Darwin Core extension ‘dwc:MeasurementOrFact’. At the time of publication, MeasurementOrFact data could not be obtained through the Global Biodiversity Information Facility (GBIF, https://www.gbif.org/), but can be downloaded via the Integrated Publishing Toolkit (IPT) link https://ipt.inbo.be/resource?r=vmm-macroinvertebrates-events.

The data are standardised to Darwin Core (Wieczorek et al. 2012) with a custom SQL view on the original VMM database and then published making use of the GBIF Integrated Publishing Toolkit (Robertson et al. 2014) instance at the INBO (http://data.inbo.be/ipt). The following list includes some of the Darwin Core terms (DCs) (http://rs.tdwg.org/dwc/terms/) used in the dataset, with additional details on the dataset content:

– language: en (English)

– license: CC_BY_4.0

– rightsholder: Flanders Environment Agency

– eventID: notation “monitoring site code : sample number”. Note: an event stands for a single sample taken at a particular time and location

– OccurrenceID: unique identifier; notation “BEVL_VMM_serial number of macro-invertebrate (mainv) observation”. BEVL: Belgium – Vlaanderen; VMM: institution acronym

– recordedBy: name of sampler(s)

– waterBody: notation “River basin ; Name(s) of the water body(-ies) ; Water body code”. River basins: Scheldt river basin, Meuse river basin

– habitat: notation “Water body type code ; Water body type name ; Physical compartment”. The typology is according to the EU Water Framework Directive. ‘Physical compartment’ distinguishes between Water body and Sediment

– datasetID: DOI 10.15468/4cvbka

– institutionCode: VMM (Vlaamse Milieumaatschappij - Flanders Environment Agency)

– datasetName: Inland water macroinvertebrate occurrences in Flanders, Belgium

– basisOfRecord: human observation

– individualCount: number of individual in the sample according to following classes: A = 1, B = 2-10, C = 11-50, D = 51-100, E = 101-1000, F = ≥1001

– dynamicProperties:

• Substrate composition: Sand, Clay-loam, Silt, Gravel-boulders, Peat, Concrete

• Substrate condition: No remarks, Leaves & branches, Oily substances, Solid waste, Plastics, Biological life

• Pool-riffle pattern: high, present, weak, absent, permanently absent

• Sinuosity: high, present, weak, absent, permanently absent

• Current: stagnant, running

• Structure Left (river bank): strengthened, natural, dug

• Structure Right (river bank): strengthened, natural, dug

• Flow: Standing/slow, Moderate, Fast

• Water Colour: No remarks, Clearly green, Clearly brown, Clearly red, Clearly grey, Clearly black, Clearly other

• Odour: No remarks, Phenol, Manure, Fuel, H2S, Sewer gases, Chlorine, Detergents, Sludge, Other

• Tidal flow: Rising tide, Slack period, Lowering tide

• Water surface condition: No visual pollution, Plant cuttings / plants, Dead fish, Oil, Floating waste, Algal foam, Duckweed, Surfactant foam, Tar, Other

• Water column condition: No visual pollution, Algal bloom, Daphnia bloom, Methane production, Flocs, Filamentous algae, Sphaerotilus, Other

• Pollution: No visual pollution, Domestic discharges, Industrial discharges, Direct agricultural discharges, Diffuse agricultural pollution, Cyanobacteria biofilm, Unknown

– measurementID: notation “monitoring site code : sample number : parameter code”. Parameter codes are according to EEA (European Environment Agency) codes and biotic index acronyms (BBI: Belgian Biotic Index, BSI: Belgian Sediment Index, MMIF: Multi-metric Macro-invertebrate Index Flanders)

– measurementType: name of the physico-chemical parameter or biological indicator

– measurementValue: figure of the physico-chemical analysis or biotic index value

– measurementUnit: unit of the physico-chemical analysis; biotic indices are unitless

– eventDate: notation “yyyy-mm-dd”

– samplingProtocol: refers to written procedures of sampling by hand net (pond net) or artificial substrates (in the case of water body monitoring), or by grab or core sampler (in the case of sediment monitoring)

– eventRemarks: “Previous weather conditions / Current weather conditions: No remarks, Heavy rainfall, Very sunny”

– scientificName: see taxonomic list

– kingdom: Animalia

– taxonRank: phylum, order, family, genus, species

– nomenclaturalCode: ICZN

– taxonRemarks: only applies to the distinction between Chironomidae *thummi-plumosus* and Chironomidae *non-thummi-plumosus*

– locationID: monitoring site code

– countryCode: BE (Belgium), FR (France), NL (the Netherlands)

– publishingCountry: BE

– continent: Europe

– municipality: name of community

– decimalLatitude: latitude of the sampling site

– decimalLongitude: longitude of the sampling site

– geodeticDatum: WGS84

– verbatimLatitude: Lambert72

– verbatimLongitude: Lambert72

– verbatimCoordinateSystem: Belgian Lambert72
